# Soluble Fas Ligand Is Essential for Blister Formation in Pemphigus

**DOI:** 10.3389/fimmu.2018.00370

**Published:** 2018-02-26

**Authors:** Roberta Lotti, En Shu, Tiziana Petrachi, Alessandra Marconi, Elisabetta Palazzo, Marika Quadri, Ann Lin, Lorraine A. O’Reilly, Carlo Pincelli

**Affiliations:** ^1^Laboratory of Cutaneous Biology, Department of Surgical, Medical, Dental and Morphological Sciences, University of Modena and Reggio Emilia, Modena, Italy; ^2^Molecular Genetics of Cancer Division, The Walter and Eliza Hall Institute of Medical Research, Parkville, VIC, Australia; ^3^Department of Medical Biology, The University of Melbourne, Parkville, VIC, Australia

**Keywords:** pemphigus, Fas ligand, apoptosis, keratinocytes, autoantibodies, cell adhesion, mouse model

## Abstract

Pemphigus is a blistering disease characterized by pemphigus autoantibodies (PVIgG) directed mostly against desmogleins (Dsgs), resulting in the loss of keratinocyte adhesion (acantholysis). Yet, the mechanisms underlying blister formation remain to be clarified. We have shown previously that anti-Fas ligand (FasL) antibody (Ab) prevents PVIgG-induced caspase-8 activation and Dsg cleavage in human keratinocytes, and that sera from pemphigus patients contain abnormally increased levels of FasL. Here, we demonstrate that recombinant FasL induces the activation of caspases prior to Dsg degradation, and anti-FasL Ab prevents acantholysis in cultured keratinocytes. Moreover, the silencing of FasL reduces PVIgG-induced caspase-8 activation and Dsg3 cleavage. Following injection of PVIgG into mice, FasL is upregulated at 1–3 h and is followed by caspase-8-mediated keratinocyte apoptosis, before blister formation. The administration of anti-FasL Ab after PVIgG injection blocks blister formation in mice. Furthermore, we injected PVIgG into two different gene-targeted mutant mice that selectively lack either secreted soluble FasL (sFasL), *FasL^Δs/Δs^* mice, or the membrane-bound form of FasL (mFasL), *FasL^Δm/Δm^* mice. After PVIgG treatment, blisters are only visible in *FasL^Δm/Δm^* animals, lacking mFasL, but still producing sFasL, similar to wild-type (C57BL/6) animals. By contrast, a significant decrease in the relative acantholytic area is observed in the *FasL^Δs/Δs^* animals. These results demonstrate that soluble FasL plays a crucial role in the mechanisms of blister formation, and blockade of FasL could be an effective therapeutic approach for pemphigus.

## Introduction

Pemphigus is a rare and potentially lethal autoimmune skin disease, characterized by the loss of keratinocyte adhesion at the level of desmosomes, a phenomenon known as acantholysis. This results in the formation of flaccid blisters and/or erosions in both skin and mucous membranes (1). Pathogenic autoantibodies [pemphigus autoantibodies (PVIgG)] in sera from pemphigus patients target predominantly molecular components of the desmosomes, such as desmoglein (Dsg)3 [pemphigus vulgaris (PV)] and Dsg1 [pemphigus foliaceous (PF)] [reviewed by Stanley and Amagai (2)]. Although autoantibodies play an essential role in the pathogenesis of pemphigus (3), the mechanisms leading to the formation of the blisters remain largely unknown (4, 5). Several lines of evidence indicate that apoptosis is involved in the pathological mechanisms of pemphigus [reviewed by Grando et al., (6)]. In particular, cleaved caspase-8 and -3 are detected in pemphigus lesions, and caspase-8-positive cells express Fas ligand (FasL)/Fas binding (7, 8).

Fas Ligand is a transmembrane protein (mFasL) that can be proteolytically cleaved to generate its soluble form of 26 kDa (sFasL) (9). Both forms of FasL can bind to their receptor, Fas, also known as CD95 or Apo1. The membrane-bound form triggers the extrinsic apoptotic pathway through the activation of caspase-8 (10), while the soluble form appears to have both apoptotic and pro-inflammatory activities in immune cells (11, 12). In healthy skin, FasL is localized in the basal layer and in the first suprabasal layers of the epidermis, homogeneously distributed within the cytoplasm, in association with intermediate filaments (13). PVIgG upregulate FasL at the mRNA and protein level in human keratinocytes (14). We have shown previously that anti-FasL antibodies (Ab) prevent PVIgG-induced caspase-8 activation and Dsg cleavage in human keratinocytes (6) and that sera from pemphigus patients contain abnormally increased levels of FasL (15).

In this study, we demonstrate that the inhibition of FasL prevents apoptosis and acantholysis *in vitro*, and anti-FasL Ab blocks blister formation *in vivo*. Finally, using two mutant mice selectively lacking the sFasL or mFasL, we provide evidence that sFasL plays a critical role in blister formation in pemphigus.

## Materials and Methods

### Pemphigus Samples

Biopsies (4-mm punch) from lesional and perilesional pemphigus skin as well as from normal skin of healthy volunteers were obtained from the Institute of Dermatology, at the University of Modena and Reggio Emilia. Samples were embedded in OCT and frozen at −20°C. In all patients, diagnosis was based on (1) typical skin and/or mucous membrane lesions, (2) detection of circulating autoantibodies by indirect immunofluorescence on monkey esophagus (a titer of 1:320), and (3) reactivity to Dsgs in patients’ sera by ELISA (MBL International Corp., Nagoya, Japan). Sera were collected from patients with PV, mucocutaneous pemphigus, and PF and pooled for IgG purification (Dsg1: 106.2 U/ml; Dsg3: 158.2 U/ml). Sera were also collected from healthy volunteers (Dsg1: 2.6 U/ml; Dsg3: 3.2 U/ml).

### IgG Purification

Pemphigus autoantibodies and normal human IgG (NIgG) were purified by affinity binding on a HiTrapProtein G HP column (GE Healthcare Bio-Science, Piscataway, NJ, USA). Sera were pooled and diluted (1:10) in 20 mM phosphate buffer (pH 7) and loaded on the column. Bound IgG was eluted with 0.1 M glycine/HCl (pH 2.7) and immediately neutralized by Tris 1 M (pH 9). Purified IgGs were dialyzed extensively against phosphate-buffered saline (PBS) (pH 7.4), concentrated by ultrafiltration (Amicon, Beverly, MA, USA), filter-sterilized, and stored at +4°C until use. Protein concentration was determined by Bradford assay using protein Standard I (BioRad, Hercules, CA, USA).

### Cell Cultures

Normal human keratinocytes were obtained from foreskin and cultured as described previously (16). Briefly, normal human keratinocytes were amplified on mitomycin C (Sigma-Aldrich, St Louis, MO, USA)-treated 3T3 cells and cultivated in Dulbecco’s modified Eagle’s medium and Ham’s F12 medium. Subconfluent secondary cultures for experiments were plated in a defined serum-free medium (KGM, Lonza Walkersville Inc., Walkersville, MD, USA). When cells were confluent, Ca^2+^ concentration was increased to 1.8 mM for 24 h, to induce keratinocyte differentiation, before the addition of any stimulus. Either human recombinant soluble FasL (rFasL) (0.1, 10, or 50 ng/ml, Sigma) or PVIgG and NIgG (1.5 mg/ml) were diluted in keratinocyte basal medium (Lonza) plus 1.8 mM Ca^2+^ and cycloheximide (Sigma) 1 µg/ml, and incubated for a further 72 h. For *in vitro* inhibitory experiments, keratinocyte cultures were pretreated with either 1 or 15 µg/ml of purified mouse anti-human FasL monoclonal Ab (NOK-2; BD Biosciences Pharmingen, San Diego, CA, USA) for 1 h, which was also added when medium was provided with NIgG, PVIgG, or rFasL.

### Passive Transfer Pemphigus Mouse Model

Neonatal C57BL/6NCrl mice (1–2 days old with body weight of approximately 1.3 g) were used for IgG passive transfer experiments [modified from Ref. (17)]. C57BL/6NCrl adult mice were obtained from Charles River (Calco, Italy) and maintained at the Laboratory Animal Facility, University of Modena and Reggio Emilia (Modena). Briefly, 5 mg/g/bw of purified PVIgG or NIgG in a total volume of maximum 50 µl was administered to neonatal mice by a single subcutaneous (s.c.) injection in the dorsal area. Twenty hours after IgG injection, animals were sacrificed and samples were collected. *FasL^Δm/Δm^* and *FasL^Δs/Δs^* animals were generated as described by O’Reilly et al. (12) and maintained at the Walter and Eliza Hall Institute of Medical Research (Melbourne) Animal Facility. FasL-mutant mice and their WT littermates were treated as described above. Direct immunofluorescence with anti-human IgG (Dako, Glostrup, Denmark) was used to demonstrate PVIgG deposition at the skin level. For time course studies, animals (*n* = 5) were sacrificed at various time points post IgG injection (0, 1, 3, 6, 9, 12, 18, and 24 h). Skin samples of each animal were harvested for hematoxylin and eosin (H&E) staining, TUNEL assay, and Western blotting. For inhibitory experiments, mice were injected s.c. with 40 µg/mouse of purified hamster anti-mouse CD178 (FasL) monoclonal Ab (MFL3; BD Pharmingen, San Jose, CA, USA) in the same dorsal area of PVIgG or NIgG (for control) injection, either 1, 2, or 3 h after IgG administration. As negative control, we used purified hamster IgG1, κ-Isotype control (40 µg/mouse; BD Pharmingen).

### Ethics Statements

For human samples, this study was carried out in accordance with the recommendations of the Ethic Committee of the IDI-IRCCS (Istituto Dermatopatico dell’Immacolata, Rome) with written informed consent from all subjects. All subjects gave written informed consent in accordance with the Declaration of Helsinki. The protocol was approved by the Ethic Committee of the IDI-IRCCS (Istituto Dermatopatico dell’Immacolata, Rome).

For WT C57BL/6 mice procedures, this study was carried out in accordance with the recommendations of the Ethical Committee of the University of Modena and Reggio Emilia and was in accordance with the Italian Institute of Health guidelines. The protocol was approved by the Italian Institute of Health. Animal studies conducted on FasL-mutant mice at the Walter and Eliza Hall Institute were approved by the Institute’s Animal Ethics Committee.

### Immunohistochemistry

Cryosections (4 µm) of skin from healthy donor and from pemphigus patients were methanol-fixed and rehydrated in PBS. The staining was performed using the UltraVision LP Detection System AP Polymer & Fast Red Chromogen assay (Thermo Fisher Scientific), according to the manufacturer’s instructions. Briefly, slides were treated with Ultra V Block, and samples were incubated with mouse anti-CD95/Fas Ab (UB2; Immunotech, Marseille, France) for 1 h at room temperature. After washes in PBS, Primary Antibody Enhancer (Thermo Fisher Scientific) was added for 20 min at room temperature, followed by incubation with AP Polymer anti-mouse/rabbit IgG for 30 min at room temperature. Slides were stained with Fast Red using Naphthol Phosphate as substrate. Samples were analyzed under a conventional optical microscope (Zeiss Axioskope 40).

### FasL siRNA Keratinocyte Transfection

About 8 × 10^4^ cells/well were plated on six-well plates in penicillin/streptomycin-free medium. After 24 h, normal human keratinocytes were transfected with 40 nM FasL siRNA (siGenome SMARTpool) or scrambled RNAi, as control (Dharmacon Inc., Lafayette, CO, USA), combined with Lipofectamine 2000 and Opti-MEM (both from Invitrogen Corporation, Carlsbad, CA, USA), according to manufacturer’s indications. Cells were transfected twice and used for PVIgG experiments. FasL protein levels were detected by Western blotting, as described below.

### Western Blotting

Cells were washed with PBS and lysed on ice in RIPA buffer (pH 7.5) containing protease inhibitors (Complete Mini Tablets, Roche). Total protein of 40 µg was analyzed on polyacrylamide gels and blotted onto nitrocellulose membranes. Blots were blocked for 2 h in a blocking buffer (5% nonfat milk in PBS/0.2% Tween20, Sigma) and incubated overnight at 4°C with the following primary Ab: anti-Dsg3 (5H10; Santa Cruz Biotechnology), anti-caspase-8 (Ab-3; Calbiochem, Darmstadt, Germany), anti-FasL (Abcam, Cambridge, UK), or anti-β-actin Ab (AC-15; Sigma). Membranes were washed in PBS/Tween, incubated with HRP-conjugated goat anti-mouse Ab (Biorad) for 45 min at room temperature, washed, and developed using the ECL chemiluminescent detection system (Amersham Biosciences UK Limited, Little Chalfont Buckinghamshire, UK). Mouse skin proteins were extracted by homogenization in SDS buffer (62.5 mM Tris-HCl pH 6.8, 2% SDS, and 10% glycerol). Proteins were separated by SDS-PAGE and transferred onto nitrocellulose membranes. Blots were blocked with 5% nonfat dry milk in TBS/0.1% Tween 20 for 1 h, then incubated overnight at 4°C with the primary rabbit polyclonal Ab mouse-specific anti-caspase-8 (Cell Signaling Technology, Danvers, MA, USA), anti-caspase-3 (Cell Signaling Technology), anti-FasL (Abcam, Cambridge, UK), or anti-β-actin Ab (AC-15; Sigma). Subsequently, membranes were incubated with the appropriate HRP-conjugated secondary Ab (Biorad) and developed, as described above. The band intensity was quantitatively determined using Image J software.

### Immunofluorescence

For double cell staining, keratinocyte cultures were washed in PBS, fixed in 4% paraformaldehyde for 20 min, and air-dried. After rehydration in PBS, cells were permeabilized for 2 min with 0.1% Triton X-100, treated for 5 min with 50 mM NH_4_Cl, and incubated with 1% bovine serum albumin for 20 min. Cells were then incubated at room temperature for 45 min with mouse monoclonal anti-Dsg3 Ab (5H10, Santa Cruz Biotechnology Inc., Santa Cruz, CA, USA) and for 45 min with Alexa Fluor 488 anti-mouse (Invitrogen). Keratinocytes were washed in PBS/Tween, labeled with rabbit polyclonal anti-caspase-3 active Ab (R&D Systems Inc., Minneapolis, MN, USA) for 45 min and with Alexa Fluor 546 anti-rabbit IgG (Invitrogen) for 45 min. For visualization of nuclei, 4',6-diamidino-2-phenylindole (DAPI) (1 µg/ml, Sigma) was used. Fluorescent specimens were analyzed by a Confocal Scanning Laser Microscopy (Leica TCS SP2). For mouse-tissue staining, paraffin-embedded tissue sections (4 µm) of skin from NIgG and PVIgG-treated mice were paraffin dewaxed, rehydrated, and antigen retrieved (citrate buffer, Thermo Fisher Scientific Inc., Fremont, CA, USA). Sections were incubated for 1 h with anti-active/cleaved caspase-8 (Novus Biologicals, Littleton, CO, USA) and for 45 min with Alexa Fluor 546 anti-rabbit IgG Ab (Invitrogen). For visualization of nuclei, 4',6-DAPI (1 µg/ml, Sigma) was used. Fluorescent specimens were analyzed by a Confocal Scanning Laser Microscopy (Leica TCS SP2).

### Dispase-Based Dissociation Assay

Normal human keratinocytes were seeded onto 12-well plates, cultured, and treated at confluency, as described above. After washing with PBS twice, cells were incubated with dispase II (>2.4 U/ml; Roche) for 30 min to release cells as monolayers. Released monolayers were carefully washed with PBS twice and subjected to mechanical stress by pipetting with a 1-ml pipetman. Fragments were fixed with 4% formaldehyde and stained with 1% Rhodamine B (Sigma). For each single well, the particle number was counted and dissociation scores were calculated using the number of fragmented cell sheets (*N*) using the following formula: dissociation score = [(N-NNIgG)/(NPVIgG-NNIgG)] × 100.

### Measurement of the Relative Acantholytic Area

The extent of epidermal acantholysis was measured microscopically by H&E staining. Samples were analyzed using a conventional optical microscope (Zeiss Axioskope 40). Five random microscopic fields per sample were captured at 200× magnification. AxioVision AC imaging software was used to acquire sample images. ImageJ software was used to count the number of pixels corresponding to the length of each cleft corresponding to the areas of the epidermis in which suprabasal cell detachment spreads along more than four adjacent basal cells. The percentage of the acantholytic area was calculated as the mean of different fields. All samples were reported as a ratio against PVIgG treatment or WT animals (in FasL-mutant mice experiments), to which a value of 1 was assigned.

### Caspase Assay

Normal human keratinocytes were grown in a multi-96 well black plate and treated with FasL (50 ng/ml). The rate of caspase-3 and -7 activation was measured simultaneously with the fluorimetric Apo-ONE Homogeneous Caspase-3/7 Assay (Promega Corporation, Madison, WI, USA), according to the manufacturer’s instructions. Briefly, Apo-ONE Caspase-3/7 reagent mixture, containing the profluorescent substrate Z-DEVD-Rhodamine 110 and lysis/permeabilization buffer, was added to each well, gently mixed, and incubated in the dark for 2 h at room temperature. After incubation, the amount of fluorescent product, which is proportional to the amount of caspase-3/7 cleavage, was measured with a FLUOstar Galaxy fluorimeter (BMG Labtech, Germany), using an excitation wavelength of 499 nm and an emission of 521 nm. Living cell number was quantified by MTT assay in a duplicate transparent plate, by incubating in MTT solution (Sigma) for 4 h at 37°C. The formazan dye produced after DMSO solubilization was evaluated by a multiwell scanning spectrophotometer at 540 nm. The final RFLU/O.D. caspase assay results were normalized in relation to MTT values from the same experiment.

### TUNEL Assay

Mouse skin samples were fixed in 4% buffered formalin and embedded in paraffin. Paraffin sections were processed for TUNEL assay using the “*In situ* cell death detection kit” (Roche Diagnostics, Basel, Switzerland), according to the manufacturer’s instructions. Briefly, following dewaxing and rehydration, sections were treated with proteinase K (20 µg/ml; Roche) for 30 min at room temperature and incubated with a reaction mixture containing TdT and fluorescence-conjugated dUTP for 1 h at 37°C. The labeled DNA was examined by a Confocal Scanning Laser Microscopy (Leica TCS SP2 with AOBS).

### Statistical Analysis

Data are presented as mean ± SEM or as ratios with group differences, obtained from three to five different experiments. Prism Software (Graph Pad Software V7.0) was used to perform statistical analysis. A two-tailed unpaired Student’s *t*-test was used for statistical comparisons between two groups, while one-way ANOVA was used for multiple comparisons (as indicated in figure legends). A value of *P* < 0.05 or less was assumed to indicate a statistically significant difference in the compared parameters.

## Results

### FasL Induces Apoptosis and Acantholysis in Human Keratinocytes

**Figure 1:**
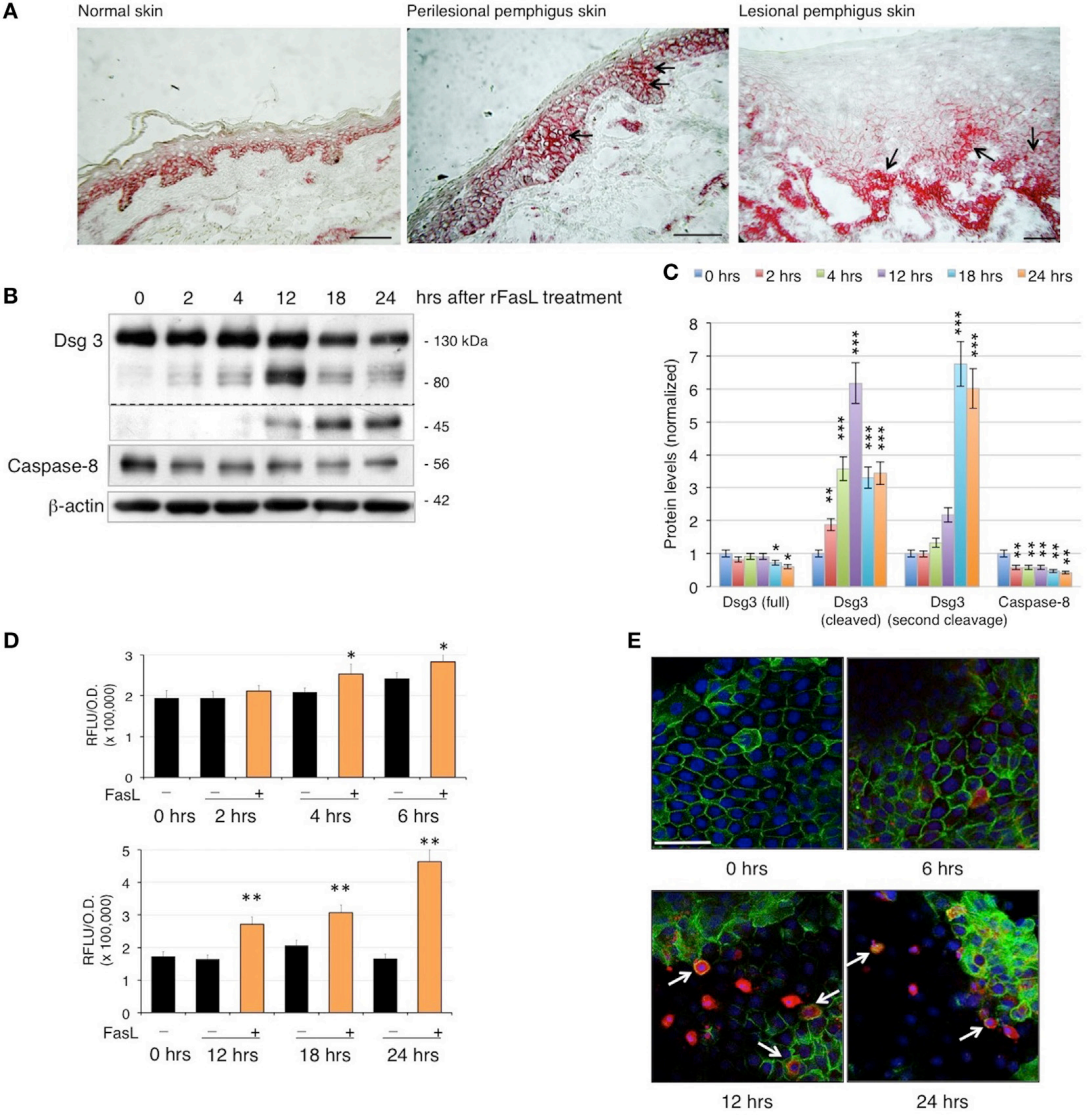
Fas ligand (FasL)-induced apoptosis precedes acantholysis in human keratinocytes: **(A)** Fas is upregulated in pemphigus skin before cell detachment. Cryosections from normal human skin, perilesional, and lesional pemphigus skin were methanol-fixed and stained with anti-Fas antibody (arrows), scale bar: 100 µm. **(B–E)** rFasL induces desmoglein (Dsg)3 cleavage through caspases activation in a time-dependent manner. Differentiated keratinocyte monolayers were treated with 50 ng/ml of recombinant soluble FasL (rFasL) for different time points. Cell lysates were **(B)** immunoblotted and **(C)** protein amounts were quantified (means ± SEM; *P < 0.05; **P < 0.01; ***P < 0.001 per Student’s *t*-test vs cntrl; *n* = 5 independent experiments). Dsg3 (full), 130 kDa; Dsg3 (cleaved), 80 kDa; Dsg3 (second cleavage), 45 kDa. **(D)** Caspase-3/7 activity was measured at each time point in the presence (+, orange bars) or absence (−, black bars) of rFasL (see Materials and Materials). Statistical analysis was performed comparing rFasL-treated samples with its cntrl (−, diluent) at each time point (means ± SEM; *P < 0.05; **P < 0.01 per Student’s *t*-test, compared with diluent; *n* = 3–5 independent experiments). **(E)** A representative immunofluorescence image shows Dsg3 (green) and active caspase-3 (red) expression in keratinocytes treated with rFasL at the indicated time points. Nuclei are shown in blue (4',6-diamidino-2-phenylindole). Scale bar = 70 µm.

Since PVIgG have been shown to induce the co-aggregation of FasL and Fas receptor with caspase-8 in death–inducing-signaling complex (7), we first analyzed the expression of Fas in pemphigus skin. While in normal epidermis, Fas expression is confined to the surface of all basal cells, in pemphigus lesions, Fas is also found in the suprabasal layers, even before cell detachment (Figure 1A). Given that PVIgG upregulate caspase-8 and caspases can cleave several adhesion molecules, including Dsgs (18, 19), we investigated the effect of recombinant soluble FasL (rFasL) on apoptosis and Dsg3. rFasL induces the cleavage of Dsg3 in a dose-dependent manner (Figure S1 in Supplementary Material) with the appearance of a cleaved 80 kD band after 12 h (Figures 1B,C), generated by the first degradation of the Dsg3 intracellular tail. In addition, rFasL induces the activation of caspase-8 commencing at 2 h (Figures 1B,C), followed by the activation of caspase-3/7 at 4–6 h (Figure 1D). An additional Dsg3 cleavage band at 45 kDa is progressively generated (Figures 1B,C), indicating further Dsg degradation induced by caspases. By confocal microscopy, cleaved (active) caspase-3-expressing cells (red) start to appear from 6 h when Dsg expression (green) is still intact. The increase in cleaved caspase-3-positive cells is associated with a reduction in Dsg expression and a progressive cell-to-cell detachment (Figure 1E, arrows). These findings suggest that the final step of the apoptotic process (e.g., activation of the executioner caspase-3) occurs in the presence of Dsg3 and before cell detachment.

**Figure 2:**
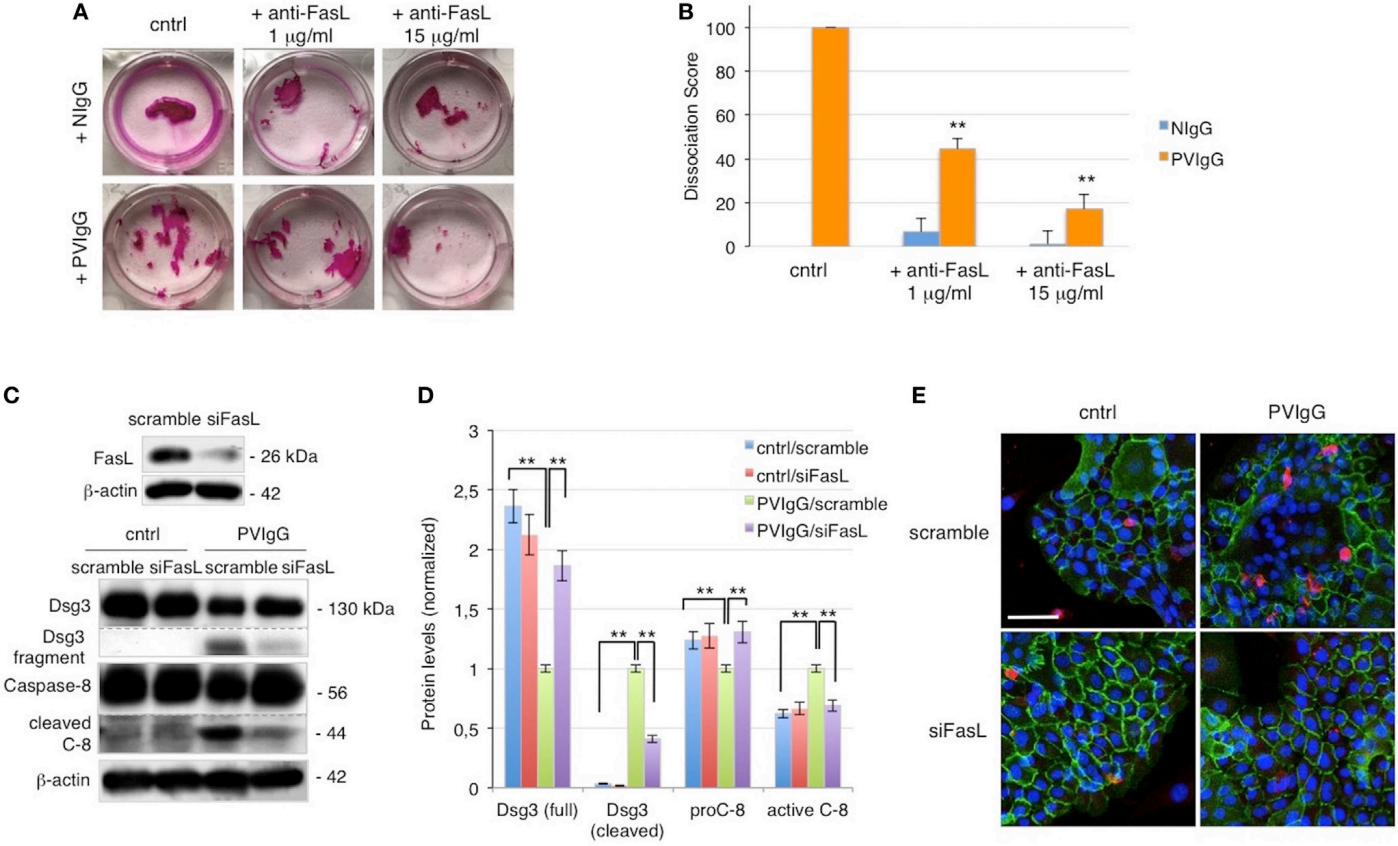
Keratinocyte-derived Fas ligand (FasL) induces desmoglein (Dsg)3, cleavage, and caspase activation. **(A,B)** Anti-FasL antibody (Ab) prevents keratinocyte acantholysis. Keratinocytes were cultured with purified normal human IgG (NIgG) or pemphigus autoantibodies (PVIgG) in the presence or absence of anti-FasL Ab (1 or 15 µg/ml), and dispase-based dissociation assay was performed. **(A)** Representative images of cell sheet fragmentation. **(B)** Quantification obtained by counting the number of fragments of cell sheets and represented as dissociation score (means ± SEM; **P < 0.01 per one-way ANOVA versus PVIgG; *n* = 5 independent experiments). **(C–E)** FasL silenced keratinocytes are protected from PVIgG-induced acantholysis and apoptosis. FasL or scrambled siRNA transfected keratinocytes were treated with PVIgG or diluent (cntrl), and levels of Dsg3 and caspase-8 were assessed by immunoblot **(C)** and measured by relative protein quantification **(D)**. **(E)** Immunofluorescence showing Dsg3 (green) and active caspase-3 (red) expression in keratinocytes. Scale bar = 70 µm.

To further evaluate the role of FasL in pemphigus, we performed the cell dissociation assay, a well-established dispase-based method to measure keratinocyte acantholysis *in vitro* (20). The addition of anti-FasL Ab clearly protects keratinocytes against acantholysis induced by PVIgG treatment, in a dose-dependent fashion (Figure 2A). Dissociation scoring shows a statistically significant reduction of cell sheet fragmentation in the presence of anti-FasL (Figure 2B). These data indicate that FasL is relevant to cell-to-cell detachment *in vitro*. In addition, the silencing of FasL using siRNA diminishes Dsg3 cleavage and the activation of caspase-8 induced by PVIgG (Figures 2C,D). The finding was further confirmed by immunofluorescence and indicated that blocking FasL by siRNA inhibits Dsg (green) the degradation and the activation of caspase-3 (red, Figure 2E). Taken together, these findings show that FasL plays a critical role in mediating PVIgG-induced apoptosis and acantholysis *in vitro*.

### Anti-FasL Ab Blocks Blister Formation *In Vivo*

**Figure 3:**
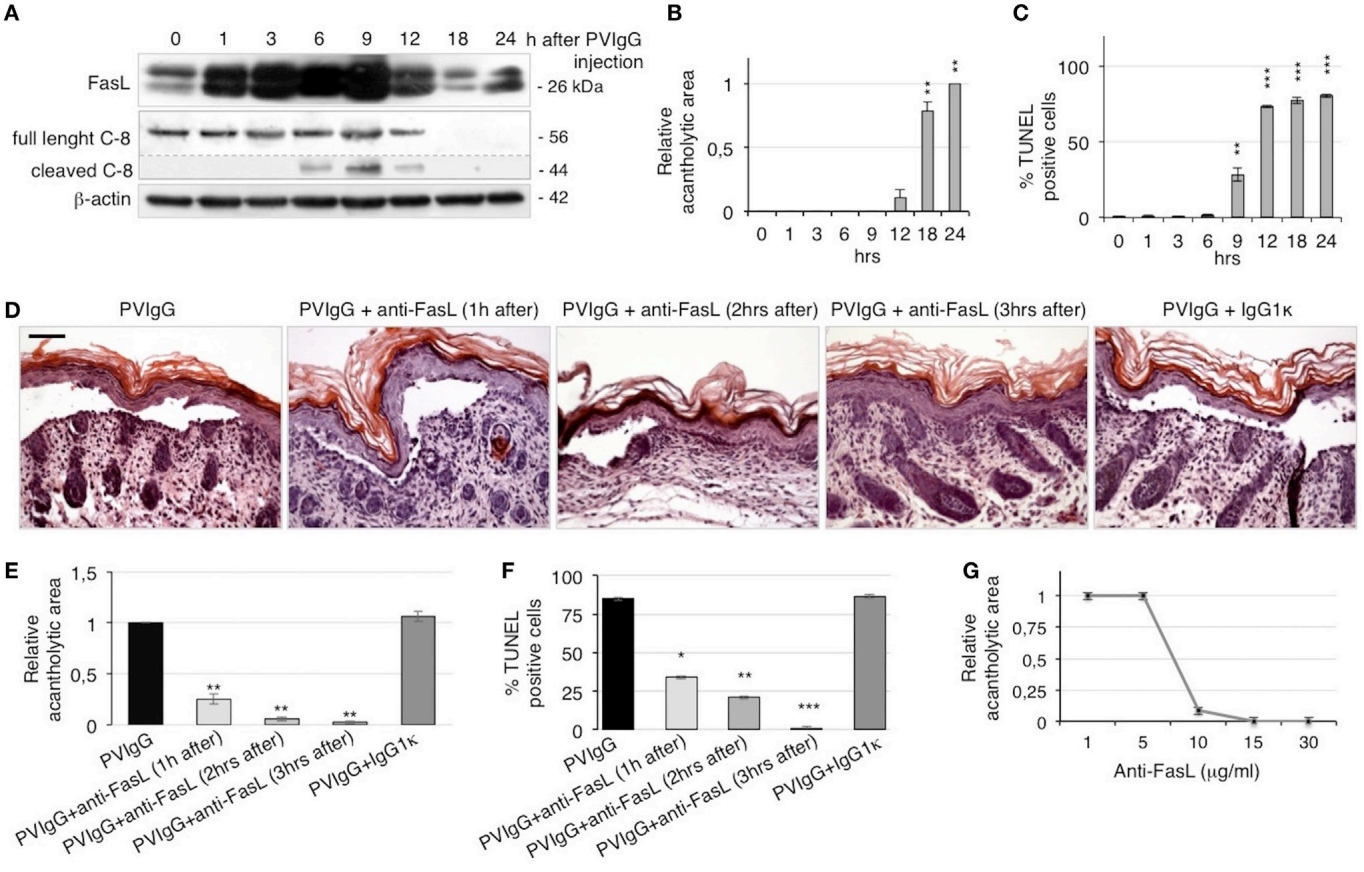
Anti-Fas ligand (FasL) antibody (Ab) inhibits blister formation in pemphigus *in vivo*. **(A–C)** Neonatal mice were injected with pemphigus autoantibodies (PVIgG), sacrificed at various time points, and analyzed for the presence of FasL, acantholysis, and apoptosis activation. **(A)** FasL protein expression and caspase-8 activation in mouse skin extracts. **(B)** Relative acantholytic areas and **(C)** the percentage of TUNEL-positive cells in mouse skin. Each time point was compared to 0 h for statistical analysis (means ± SEM; **P < 0.01; ***P < 0.001 per one-way ANOVA; *n* = 5 animals/each time point). **(D–F)** Neonatal mice were injected with normal human IgG (NIgG) or PVIgG, treated with anti-FasL Ab at the indicated time points and sacrificed at 20 h post PVIgG injection. IgG1κ was used as an isotype control for the anti-FasL Ab. **(D)** Hematoxylin and eosin staining of the neonatal mouse skin. Scale bar = 100 µm. **(E)** Relative acantholytic areas in mouse skin and **(F)** the percentage of TUNEL-positive cells in mouse skin (means ± SEM; *P < 0.05; **P < 0.01; ***P < 0.001 per Student’s *t*-test, compared with PVIgG; *n* = 5 animals/each treatment). **(G)** Mice were treated with PVIgG and, 3 h later, with decreasing doses of anti-FasL Ab. Relative acantholysis areas were measured in at least three different fields (*n* = 5 animals/each treatment).

To confirm the role of FasL in the pathological mechanisms of pemphigus *in vivo*, we s.c. injected neonatal C57BL/6N mice with purified pathogenic PVIgG or NIgG. Twenty hours later, the intraepidermal blister and the deposition of autoantibodies at the floor and the roof of the cleft (Figure S2A in Supplementary Material) recapitulate the immuno-histologic alterations of pemphigus in humans. In addition, we detected the activated form of caspase-3 in the skin of PVIgG-treated mice but not those injected with NIgG, as shown by the appearance of the cleaved form of caspase-3 (Figure S2B in Supplementary Material). Our findings indicate that PVIgG can activate the apoptotic process *in vivo*. In particular, PVIgG can induce the upregulation of FasL protein in mouse epidermis starting at 1 h and caspase-8 activation at 6 h post injection, respectively (Figure 3A). While blisters, as measured by the relative acantholytic areas, were observed at 12 h (Figure 3B), TUNEL-positive epidermal cells were already detected from 9 h after PVIgG treatment (Figure 3C). These results demonstrate that pathogenic autoantibodies first induce the release of FasL, followed by the appearance of apoptotic epidermal cells, which in turn precedes the onset of blisters *in vivo*.

To further define the role of FasL *in vivo*, mice were injected with PVIgG alone or in combination with a FasL-blocking Ab (40 μg/mouse). Control mice that received PVIgG alone developed skin lesions 20 h after injection. By contrast, when anti-FasL Ab was administered 1 or 2 h after PVIgG injection, blisters were still visible but of lesser magnitude. Further, when anti-FasL Ab was administered 3 h after PVIgG injection, no blister formation was observed (Figure 3D), indicating that blocking FasL prevents acantholysis *in vivo*. The protective effect of anti-FasL Ab against acantholysis was confirmed by measuring the relative acantholytic areas in multiple fields from all skin specimens. The relative acantholytic area was significantly decreased when anti-FasL Ab was given 1 h after PVIgG, almost undetectable at 2 h and not measurable in mice treated with PVIgG followed by the administration of anti-FasL Ab, 3 h later (Figure 3E). Moreover, the percentage of TUNEL-positive cells was significantly higher in the epidermis from mice treated with PVIgG alone, compared to mice treated with anti-FasL Ab at 1 or 2 h after PVIgG. No TUNEL-positive cells were detected in the epidermis of mice that received anti-FasL Ab, 3 h after PVIgG (Figure 3F). Mice were subsequently treated with decreasing amounts of anti-FasL Ab which inhibited blister formation in a dose-dependent manner, as measured by the relative acantholytic area (Figure 3G).

### Soluble FasL Is Required for Blister Formation in Pemphigus

**Figure 4:**
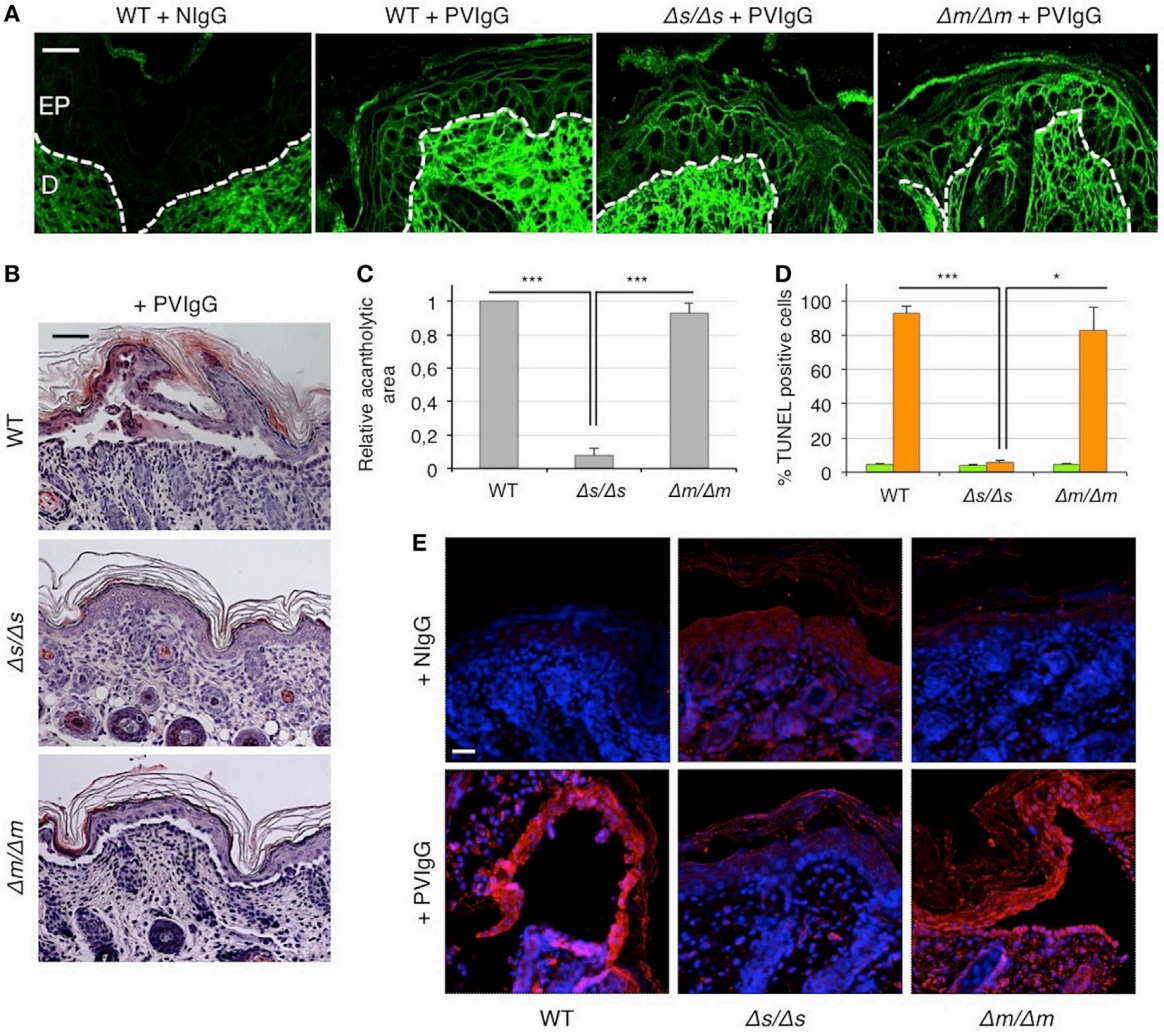
Blister formation is blocked in mice selectively lacking soluble Fas ligand (FasL). Wild-type (WT), FasLΔ*^s/Δs^*, and FasLΔ*^m/Δm^* mice were each injected with either normal human IgG (NIgG) or with pemphigus autoantibodies (PVIgG) and sacrificed at 20 h after injection. FasLΔ*^s/Δs^*: mice selectively lacking the soluble FasL; FasLΔ*^m/Δm^*: mice selectively lacking the membrane-bound FasL. **(A)** Representative images of PVIgG binding at the inter-keratinocyte level in the indicated mouse strains. Direct immunofluorescence: mouse skin specimens were challenged against fluorescein-conjugated human IgG. EP, Epidermis; D, dermis. Scale bar = 100 µm. NIgG (normal IgG) used as a negative control. **(B)** Representative hematoxylin and eosin staining of mouse skin. Scale bar = 75 µm. **(C)** Relative acantholytic areas in mouse skin (means ± SEM; ***P < 0.001 per one-way ANOVA; *n* = 10–11 mice/strain). **(D)** The percentage of TUNEL-positive cells in mouse skin treated either with NIgG (green bars) or with PVIgG (orange bars) (means ± SEM; *P < 0.05; ***P < 0.001 per one-way ANOVA; *n* = 10–11 mice/strain). **(E)** A representative immunofluorescence image showing active caspase-8 (red) expression at the skin level of NIgG or PVIgG-treated mice. Nuclei are shown in blue (DAPI). Scale bar = 100 µm.

To definitely assess the role of FasL in the mechanisms underlying pemphigus, we injected PVIgG into two different gene-targeted mutant mice that selectively lack either the secreted soluble FasL (sFasL), *FasL^Δs/Δs^* mice, or the membrane-bound FasL (mFasL), *FasL^Δm/Δm^* mice (12). Twenty hours after PVIgG treatment, despite the deposition of human IgG at the inter-keratinocyte level (Figure 4A), no acantholysis was detected in *FasL^Δs/Δs^* animals, as determined by the histological examination of H&E-stained sections (Figure 4B) and the measurement of the relative acantholytic areas (Figure 4C). By contrast, blisters were clearly visible in PVIgG-treated *FasL^Δm/Δm^* animals, lacking mFasL and in wild-type (WT) (C57BL/6) mice (Figure 4B). The magnitude of the acantholytic areas was significantly decreased (not detectable) in *FasL^Δs/Δs^* animals, as compared to that in control and *FasL^Δm/Δm^* mice (Figure 4C). Moreover, TUNEL-positive cells were statistically increased only in the skin of PVIgG-treated WT and *FasL^Δm/Δm^*, but not in *FasL^Δs/Δs^* animals (Figure 4D). Finally, PVIgG injection failed to induce the activation of caspase-8 only in *FasL^Δs/Δs^* animals (Figure 4E).

## Discussion

Pemphigus is caused by autoantibodies targeting keratinocyte surface antigens. PVIgG binding to these proteins has long been considered to directly induce blister formation (21). Yet, it has now become clear that additional mechanisms following Ab binding contribute to skin blistering in pemphigus. A number of studies have provided evidence that reduced desmosomal adhesion and splitting follow changes in desmosomal structure and Dsg depletion (5). Moreover, several signaling pathways downstream of Ab binding, including p38 mitogen-activated protein kinase, EGFR, c-Myc, etc., have been shown to be involved in the loss of keratinocyte adhesion in pemphigus (22). In this context, the role of apoptosis has been the subject of much controversy. *In vitro* studies claim that apoptosis is not a prerequisite for skin blistering but can contribute to the acantholytic process (23). By contrast, apoptotic cells are detected before blister formation, and the inhibition of caspase 3/7 prevents intraepidermal blistering in a murine pemphigus model (24). Most importantly, ST18 overexpression in pemphigus skin (25) confers a significant risk for the disease by both upregulating apoptosis and disrupting keratinocyte adhesion (26).

Our results clearly indicate a critical role of the Fas/FasL-induced extrinsic apoptotic pathway in the pathogenesis of pemphigus, consistent with previous studies showing the expression and the activation of Fas and FasL in acantholytic cells (7, 8). Specifically, we show for the first time that Fas/FasL system is operational before blister formation. Upon binding to pemphigus antigens, the Fas/FasL system leads to the activation of apoptosis and acantholysis. Moreover, we provide evidence that apoptosis precedes acantholysis, as Fas overexpression, caspase activation, and the appearance of TUNEL-positive cells occur before cell detachment *in vivo*.

Pemphigus autoantibodies upregulate FasL at the mRNA and protein level in human keratinocytes *in vitro* (14). Here, we report that the exposure to PVIgG results in the rapid release of soluble FasL *in vivo*. Deposits of FasL are present in association with intermediate filaments in keratinocytes (13). Because keratins control intercellular adhesion (27) and their retraction is associated with signaling pathways in pemphigus (28), we speculate that PVIgG-induced keratin uncoupling from the desmosomal complex may contribute to the rapid mobilization and release of FasL, resulting in the upregulation of FasL in the intercellular milieu. Altogether, our results demonstrate that pathogenic autoantibodies first induce the release of FasL, followed by the appearance of apoptotic epidermal cells, which in turn precedes the onset of blisters *in vivo*.

The addition of an anti-FasL-neutralizing Ab blocks blister formation in mice at 1–3 h after PVIgG injection. This is consistent with the timing of FasL upregulation in the epidermis, strongly indicating that anti-FasL blocks its target when it is locally released from keratinocytes, upon PVIgG stimulation.

Because mice lacking soluble FasL fail to develop blister upon PVIgG injection, we conclude that sFasL is responsible for acantholysis *in vivo*. The data presented here strongly indicate sFasL activation of caspase-8 and subsequent activation of caspase-3 as the main mechanism accounting for Dsg cleavage and blister formation in pemphigus. However, soluble FasL does not only trigger the extrinsic apoptotic pathway but also exert a number of pro-inflammatory activities (29, 30), which may be involved in pathological mechanisms of pemphigus. Whether these inflammatory responses could contribute to the development of blister remains to be determined.

Previous studies have attempted to prevent blister formation by pretreating mice with various signal transduction inhibitors, before PVIgG injection (31, 32). By contrast, in the present study, anti-FasL Ab blocks blister formation by acting after PVIgG injection, thus functioning in a system that is closely analogous to human disease. Currently, pemphigus therapy consists of the chronic administration of steroids and other immunosuppressors, which often results in severe side effects with a high fatality incidence. Blocking FasL-induced blister formation provides a relevant proof of concept that may lead to the development of new drugs to ameliorate pemphigus, possibly replacing the use of broad immunosuppressive agents.

## Author Contributions

RL, AM, and CP designed the studies; RL, ES, and TP conducted *in vivo* experiments on WT animals; AL and LO conducted *in vivo* experiments on FasL-mutant mice; RL, MQ, and EP conducted *in vitro* experiments; RL and AM analyzed data; RL, LO, and CP wrote the manuscript.

## Conflict of Interest Statement

CP and AM are cofounders of PinCell s.r.l., a start-up company involved in drug development for pemphigus. They are coinventors on a patent that includes some data presented in this work (WO2010066914A3). Other authors declare no competing financial interests.
