# Supplementary material for: Soluble Fas Ligand Is Essential for Blister Formation in Pemphigus
Source: Front Immunol. 2018 Feb 26;9:370. doi: 10.3389/fimmu.2018.00370 (PMC5834757; doi:10.3389/fimmu.2018.00370)
Supplement: Supplementary file 1 [file presentation_1.pptx]

## Slide 1
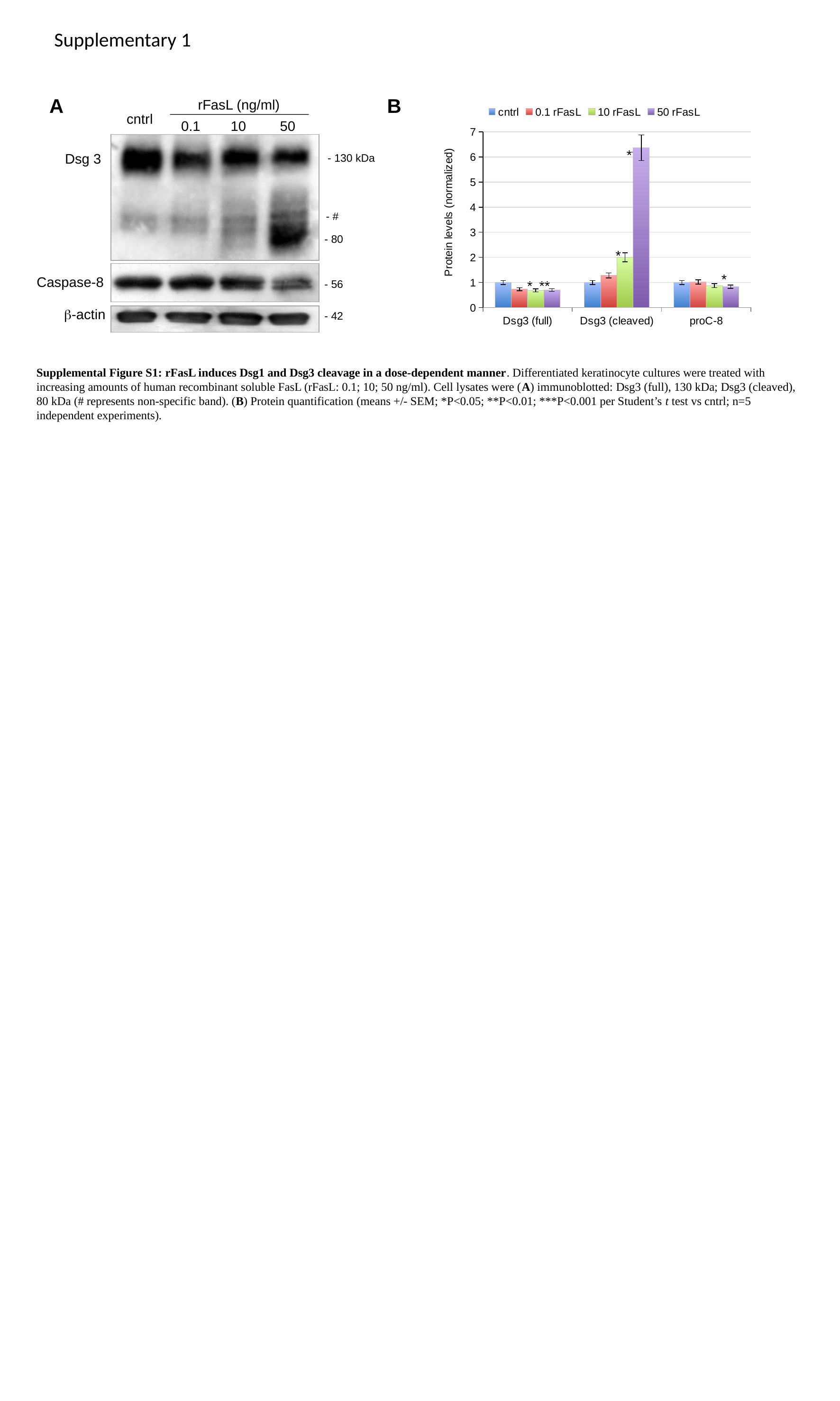

# Supplementary 1
A
B
rFasL (ng/ml)
### Chart
| Category | cntrl | 0.1 rFasL | 10 rFasL | 50 rFasL |
|---|---|---|---|---|
| Dsg3 (full) | 1.0 | 0.730623656887682 | 0.683284812746463 | 0.696313084230942 |
| Dsg3 (cleaved) | 1.0 | 1.277926238365469 | 2.00609668761368 | 6.3719183056043 |
| proC-8 | 1.0 | 1.02054832000126 | 0.870474257703209 | 0.834296406858777 |cntrl
0.1
10
50
***
Dsg 3
- 130 kDa
- #
- 80
*
*
Caspase-8
- 56
**
*
b-actin
- 42
Supplemental Figure S1: rFasL induces Dsg1 and Dsg3 cleavage in a dose-dependent manner. Differentiated keratinocyte cultures were treated with increasing amounts of human recombinant soluble FasL (rFasL: 0.1; 10; 50 ng/ml). Cell lysates were (A) immunoblotted: Dsg3 (full), 130 kDa; Dsg3 (cleaved), 80 kDa (# represents non-specific band). (B) Protein quantification (means +/- SEM; *P<0.05; **P<0.01; ***P<0.001 per Student’s t test vs cntrl; n=5 independent experiments).

## Slide 2
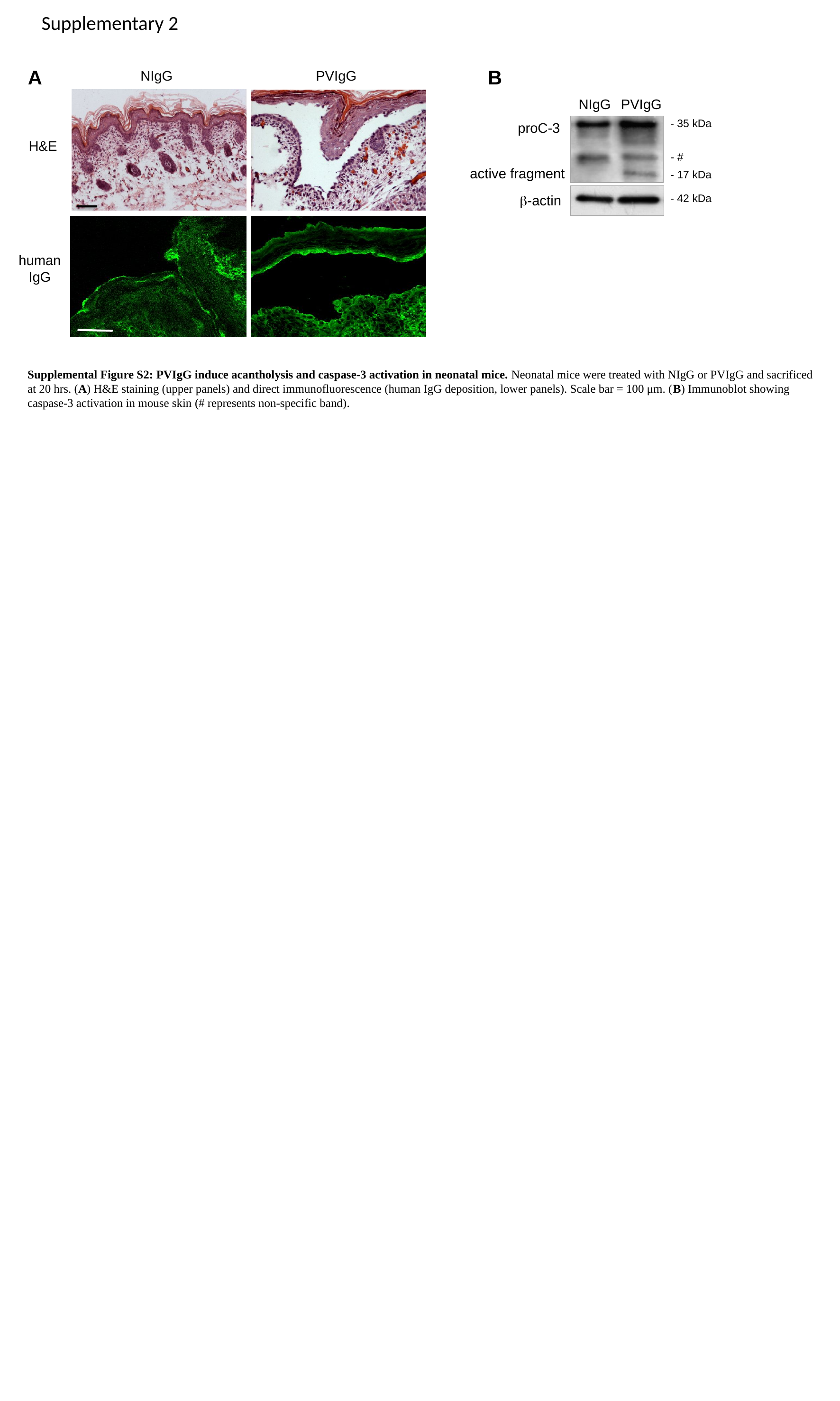

# Supplementary 2
A
B
NIgG
PVIgG
NIgG
PVIgG
- 35 kDa
proC-3
H&E
- #
- 17 kDa
active fragment
- 42 kDa
b-actin
IHC
human IgG
Supplemental Figure S2: PVIgG induce acantholysis and caspase-3 activation in neonatal mice. Neonatal mice were treated with NIgG or PVIgG and sacrificed at 20 hrs. (A) H&E staining (upper panels) and direct immunofluorescence (human IgG deposition, lower panels). Scale bar = 100 μm. (B) Immunoblot showing caspase-3 activation in mouse skin (# represents non-specific band).
